# Smart Ambient Bright Light in Nursing Homes: Impact on Individual Light Exposure and Dementia‐related Symptoms

**DOI:** 10.1002/alz70861_108600

**Published:** 2025-12-23

**Authors:** Ying‐Ling Jao, Yo‐Jen K Liao, Diane Berish, Julian Wang

**Affiliations:** ^1^ Pennsylvania State University, University Park, PA USA

## Abstract

**Background:**

Nursing home residents with dementia often experience insufficient exposure to appropriate lighting, which can disrupt circadian rhythms and exacerbate neurobehavioral symptoms. While previous research has shown that bright light during daytime helps alleviate neurobehavioral symptoms, such as depression and agitation, most studies have evaluated lighting interventions without considering individual variations in actual light exposure. Light exposure can vary considerably for residents within the same facility due to differences in their daily routines, locations, and activity patterns. Wearable light sensors offer a promising approach to accurately measure individual light exposure.

**Method:**

This study evaluated the effect of the Smart Ambient Bright Light (SABL) system on neurobehavioral symptoms for nursing home residents with dementia while tracking individual light exposure. The SABL system incorporated natural daylight and automatically delivered bright light during the day and dim light at night to support circadian health. This was a 13‐week crossover clustered randomized controlled trial with a four‐week SABL intervention and a four‐week control (usual lighting) period, with the order randomly assigned. Participants were 27 residents with dementia and agitation recruited from two nursing homes in Pennsylvania. Light exposure was measured using the LYS button, clipped on the participant's shoulder, and worn 24/7 every other week. Neurobehavioral symptoms were assessed using the Neuropsychiatry Inventory.

**Result:**

Participants, on average, were 87.4 years old and predominantly female (74%). During the intervention, participants' average light exposure was 406.9 lux during the day and 94.5 lux at night. In the control period, average light exposure was 286.6 lux during the day and 156.1 lux at night. Higher daytime light exposure was significantly associated with lower levels of apathy (*p* <0.05), appetite changes (*p* <0.01), delusions (*p* <0.05), and hallucinations (*p* <0.05). Additionally, lower nighttime light exposure was significantly associated with lower levels of agitation (*p* <0.05) and nighttime behaviors (*p* <0.01).

**Conclusion:**

The findings suggest that the SABL helps optimize light exposure and that improved exposure can mitigate certain neurobehavioral symptoms in residents with dementia. Future research should evaluate the broader implementation of SABL and its long‐term effect on a larger scale to inform best practices in environmental design and dementia care in nursing homes.

